# Attenuated Phenotype and Immunogenic Characteristics of a Mutated Herpes Simplex Virus 1 Strain in the Rhesus Macaque

**DOI:** 10.3390/v10050234

**Published:** 2018-05-02

**Authors:** Shengtao Fan, Xingli Xu, Yun Liao, Yongrong Wang, Jianbin Wang, Min Feng, Lichun Wang, Ying Zhang, Zhanlong He, Fengmei Yang, Nigel W. Fraser, Qihan Li

**Affiliations:** 1 Yunnan Key Laboratory of Vaccine Research and Development on Severe Infectious Diseases, Institute of Medical Biology, Chinese Academy of Medical Sciences & Peking Union Medical College, Kunming 650118, China; fst@imbcams.com.cn (S.F.); xinglixu@imbcams.com.cn (X.X.); liaoyun@imbcams.com.cn (Y.L.); 18669098870@163.com (Y.W.); rong122419@163.com (J.W.); fengmin@imbcams.com.cn (M.F.); wlc@imbcams.com.cn (L.W.); zhangy@imbcams.com.cn (Y.Z.); 2 Institute of Medical Biology, Chinese Academy of Medical Sciences & Peking Union Medical College, Kunming 650118, China; hzl@imbcams.com.cn (Z.H.); yangfenmei@imbcams.com.cn (F.Y.); 3 Department of Microbiology, Perelman School of Medicine, University of Pennsylvania, Philadelphia, PA 19104, USA; nfraser@upenn.edu

**Keywords:** HSV-1, mutant strain, immunogenicity, rhesus macaques

## Abstract

Herpes simplex virus type 1(HSV-1) presents a conundrum to public health worldwide because of its specific pathogenicity and clinical features. Some experimental vaccines, such as the recombinant viral glycoproteins, exhibit the viral immunogenicity of a host-specific immune response, but none of these has achieved a valid epidemiological protective efficacy in the human population. In the present study, we constructed an attenuated HSV-1 strain M3 through the partial deletion of *UL7, UL41*, and the latency-associated transcript (*LAT*) using the CRISPR/Cas9 system. The mutant strain exhibited lowered infectivity and virulence in macaques. Neutralization testing and ELISpot detection of the specific T-cell responses confirmed the specific immunity induced by M3 immunization and this immunity defended against the challenges of the wild-type strain and restricted the entry of the wild-type strain into the trigeminal ganglion. These results in rhesus macaques demonstrated the potential of the attenuated vaccine for the prevention of HSV-1 in humans.

## 1. Introduction

Studies of the herpes simplex virus (HSV) in the fields of clinical medicine and epidemiology during the past two decades have revealed that HSV-1 and HSV-2 constitute a threat to public health worldwide due to their specific pathogenicity and clinical features [1,2]. The absence of specific preventive measures and the mode of viral spreading in populations have resulted in infectious rates of 25–60% in populations of various ages worldwide [3,4], and few medicines are available for clinical treatment [5]. Progress in bioengineering technology and epidemiological observations have resulted in the development of vaccines against HSV-1 and HSV-2, and these vaccines, which were designed as recombinant viral glycoproteins associated with certain adjuvants and which demonstrated viral immunogenicity capable of stimulating a host-specific immune response, have been evaluated in clinical trials [6,7,8,9,10]. These vaccines exhibited reliable safety in children, despite the absence of a comprehensive understanding of HSV pathogenesis with characteristic latent infection, and underwent further clinical evaluation [9]. However, the data from these clinical trials did not suggest an epidemiological protective efficacy against the HSV infection in various populations [10,11]. Nevertheless, the results suggested the potential for studies on the development of replication-defective viral strains and attenuated strains with mutated genes as candidates for experimental HSV vaccines [12,13]. Although these viruses have produced interesting data in animal tests, the potential pathological risk of the HSV attenuated viral strain as a candidate vaccine, including latent infection, must be more thoroughly studied [14]. Previous data on HSV-1 and HSV-2 have demonstrated that safety evaluations of the attenuated HSV strain as a vaccine candidate were less conclusive than those of other viruses [15,16,17]. The successful application of the varicella-attenuated vaccine strain Oka suggested that an attenuated HSV strain with certain genomic mutations might be a promising vaccine candidate despite our limited knowledge of its viral pathogenesis [18,19]. In this context, studies focused on certain viral genes and their encoding proteins, which are capable of interacting with host molecules, are needed to further evaluate the attenuated phenotypes of HSV strains with mutated genes. More data on viral molecules and their effects on hosts will provide evidence for the construction of attenuated strains using artificial mutation technology. Recent studies of the HSV strains with modified or mutated genes showed an attenuation of the phenotype of the strains, supporting the prospect of an attenuated HSV vaccine [20,21].

Our previous study focused on the functional tegument proteins and latency-associated transcript (*LAT*) of HSV-1, which are associated with viral pathogenesis and latency establishment and maintenance, respectively. We constructed a series of mutated strains, including a strain that exhibited an attenuated phenotype [22]. The gene encoding tegument *UL7* was partially deleted in this mutated strain, named M1. This mutant HSV-1 showed a reduced viral proliferative rate in cultured cells and decreased virulence in mice by modulating the viral transcription of the genes [22]. The mutant M3 strain, which was based on M1, was obtained via mutations in the *UL7*, *UL41*, and *LAT* genes and exhibited an attenuated phenotype in mice [23]. However, with the biological difference and as a non-natural host for HSV-1, the mouse is not thought to be capable of providing data for the real process of the viral interactions with human tissues, especially for pathological events occurring during the latent phase [24]. The non-human primate, with the advantage of a closer genetic relationship with humans, is recognized as a better model for some viral diseases and was used in our previous work for HSV-1 infection, which suggested that this model showed positive clinical features under the viral infection [25]. It is expected that the evaluation of immunogenicity and the attenuated characteristics of M3 in rhesus macaques might establish the optimal indicators for further biological appraisal of M series mutants, which could provide candidates for a vaccine study. The present study analyzed the attenuated phenotype of M3 compared with wild-type HSV-1 in rhesus macaques and the results demonstrated that this mutant produced lower infectivity and virulence in macaques, which were asymptomatic and presented no viral proliferation in various organs one year after viral inoculation. Notably, the viral *LAT* gene was not detected in the trigeminal ganglion (TG) and immunological detection suggested an immune response, including the presence of a potential neutralizing antibody and IFN-γ-specific T cell immunity against HSV-1. Furthermore, viral infection by challenge with the wild-type strain was restricted in the inoculated macaques.

## 2. Materials and Methods

### 2.1. Preparation of the M3 Strain

The M3 mutated strain was constructed via partial deletion of the *UL7*, *UL41*, and *LAT* genes according to the methods described in our previous work [23]. In this study, the M3 virus that proliferated in Vero cells was identified again by specific PCR detection of the deleted sequences of *UL7*, *UL41*, and *LAT* genes, and the viral titer was determined in a microtitration assay [23].

### 2.2. Rhesus Administration and Ethics Statement

The animals used in this study were handled according to the Guide for the Care and Use of Laboratory Animals by the National Research Council of the National Academies [26] and the Guidelines for Experimental Animal Welfare and Ethical Treatment provided by the Ministry of Science and Technology of the People’s Republic of China (2006). The Yunnan Provincial Experimental Animal Management Association (Approval no. SCXK [Dian]K2015-0006, 1 December 2015–1 December 2020) and the Experimental Animal Ethics Committee of the Institute (Approval no. YISHENGLUNZI [2016]4, 1 April 2016) approved the experimental protocols. The animals were reared in the primate animal center of the Institute of Medical Biology (IMB) at the Chinese Academy of Medicine Science (CAMS) and were maintained under full veterinary care. All animals were isolated for 2 weeks and were confirmed negative for the herpes simian B virus infection and anti-HSV-1 antibodies [25].

### 2.3. Rhesus Macaque Experimental Design and Sample Collection

**Figure 1:**
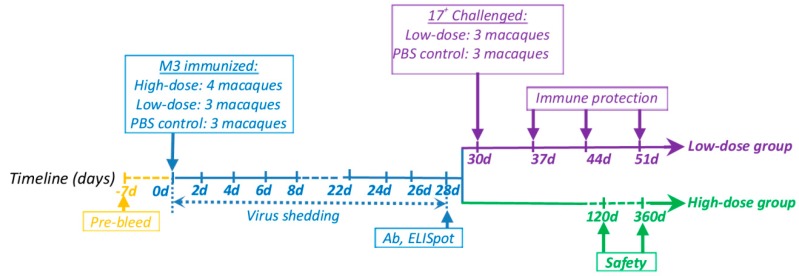
The schematic overview of the experimental process.

Seven 1.5-year-old monkeys were divided into two groups. The high-dose group included four monkeys that received 5 × 10^5^ TCID_50_ of M3 via intramuscular injection and the low-dose group consisted of three monkeys that received an intramuscular injection of 5 × 10^4^ TCID_50_ of M3 (Figure 1). The control group comprised of three monkeys that received phosphate-buffered saline (PBS). Swab samples were collected from the mouth, eye, nose, feces, and urine of the immunized monkeys continuously for 28 days after immunization and centrifuged at 8000× *g* for 8 min. The supernatants were used for q-PCR detection. All the macaques were subjected to neutralizing antibody assays and ELISpot detection on day 28, post-immunization. Two macaques from the high-dose group were anesthetized using ketamine (10 mg/kg of body weight, Phoenix Pharmaceuticals, St. Joseph, MO, USA) and sacrificed on day 120 (#16209 and #16065) or 360 (#16061 and #16137) post-immunization. Tissues from the sacrificed animals were homogenized using a Tissue Lyser II system (Qiagen, Hilden, Germany) and used for q-PCR detection. The TG was co-cultured with Vero cells and used for in situ hybridization. The low-dose and PBS control groups were scarified on the lip using a 24-gauge needle and 10^4^ TCID_50_ of the wild-type strain (17^+^) was applied. Swab samples from the infected monkeys were collected continuously for 21 days. One macaque from each group was anesthetized using ketamine (10 mg/kg of body weight, Phoenix Pharmaceuticals, St. Joseph, MO, USA) and sacrificed on day 7 (#16149 and #16173), 14 (#16175 and #16153), and 21 (#16319 and #16379), post-infection. Two macaques (#14139 and #14067) were treated by scarifying the lip as described above as the wild type (WT) control group. Various tissues were used for q-PCR detection and the TG was co-cultured with Vero cells and used for in situ hybridization.

### 2.4. q-PCR Detection of the Viral Load in Various Tissues

The viral DNA from various samples was extracted using the TaKaRa MiniBEST Viral RNA/DNA Extraction Kit Ver.5.0 (TaKaRa, Tokyo, Japan) according to the manufacturer’s instructions. q-PCR quantification was performed using pre-mix Ex Taq (probe q-PCR) (TaKaRa, Tokyo, Japan) with a BIO-RAD iCycler Thermal Cycler (Bio-Rad Laboratories, Inc., Hercules, CA, USA). The primers and probe for the detection of UL30 in this study were previously reported [27]. The primers and probe were as follows: forward primer 5′-CATCACCGACCCGGAGAGGGAC-3′, reverse primer, 5′-GGGCCAGGCGCTTGTTGGTGTA-3′, and probe 5′-CCGCCGAACTGAGCAGACACCCGCGC-3′. Samples that read five or fewer copy numbers were reported as negative.

### 2.5. In Situ Hybridization of the LAT mRNA in the TG

The RNA probes (5′-CATAGAGAGCCAGGCACAAAAACAC-3′) labeled with digoxigenin-11-UPT (DigU) used in this study were described previously [28] and an enhanced Sensitive ISH Detection Kit I (POD) (Boster, Wuhan, China) was used to detect the *LAT* mRNA, as described previously [25]. Briefly, the collected samples were fixed in 4% paraformaldehyde and treated with pepsin to expose the *LAT* mRNA. The slides were treated with the pre-hybridization solution for 4 h at 37 °C followed by hybridization in a hybridization solution with *LAT* probes at 37 °C overnight. After incubation with the anti-digoxigenin antibody conjugated to biotin for 2 h at 37 °C, the slides were treated with 3, 3′-diaminobenzidine (DAB). *LAT* positivity was visualized via light microscopy (Nikon, Tokyo, Japan).

### 2.6. Histopathological Detection

The TG samples were fixed in 10% (*w*/*v*) formalin for 7 days, dehydrated, embedded in paraffin, and then sliced for hematoxylin and eosin (HE) staining assays. Images were acquired using a light microscope (Nikon, Tokyo, Japan).

### 2.7. Co-Culture of TG and Vero Cells

The TG was removed from the sacrificed monkeys on various days post-immunization (120 and 360) or post-infection (7, 14, and 21), cut into smaller sections, and placed on monolayers of Vero cells. The cytopathic effects (CPEs) were monitored daily for 7 days. If a CPE was observed, the culture medium was harvested and DNA was extracted for HSV-1 detection. If no CPEs were detected in culture, the cells were harvested and continuously blind passaged for three generations.

### 2.8. Serum Antibody Detection by ELISA

ELISA plates (Corning, Shanghai, China) were coated with whole HSV-1 (F strain) as the antigen for serum IgG antibody detection. Briefly, Vero cells were infected with HSV-1 for 36 h, the cell suspension was harvested and sonicated for 30 s and 45 μg of cell lysate/well was coated on the ELISA plates at 4 °C overnight. The plates were washed 5 times with PBS/0.05% Tween 20 buffer and then blocked with PBS/5% BSA at 4 °C overnight. Each sample of the rhesus macaque serum was diluted at 1:500 in PBS/0.5% BSA, added to two wells, and then incubated at room temperature for 2 h. Next, the plates were washed as described above and incubated with HRP-conjugated mouse anti-rhesus IgG (Bioss, Shanghai, China) diluted at a ratio of 1:5000 in PBS /0.5% BSA at 37 °C for 1 h. TMB Single-Component Substrate solution (Solarbio, Beijing, China) was added to each well after 5 washes. The color reaction was stopped using ELISA Stop Solution (Solarbio, Beijing, China). Subsequently, the wells were read at an absorbance of 450 nm. Similarly, the ELISA plates coated with Vero cells were mock-infected as the control and the procedures were repeated as described above. The resulting values were determined by subtracting the values obtained for uninfected cell lysates from the values obtained with infected cell lysates [29].

### 2.9. Serum Neutralizing Antibody Assay

The rhesus macaque serum samples were collected and then subjected to 2-fold serial dilutions. All sera were incubated with 10^2^ TCID_50_ of the 17^+^ strain for 1 h at 37 °C. The viral titers were determined by a plaque assay using Vero cells at 37 °C in a 5% CO_2_ incubator. The end-point neutralization titer was determined using 50% plaque reduction assays.

### 2.10. ELISpot Detection of Cell-Mediating Immune Responses in Macaques

Peripheral blood mononuclear cells (PBMCs) were collected from two groups of monkeys on day 28 and treated as previously described [30]. The peptides derived from various genes (gD, gG, gB, gE) used to stimulate PBMCs were previously described [25]. Enzyme-Linked ImmunoSpot (ELISpot) assays were performed to detect the interferon (IFN)-γ concentration according to the manufacturer’s instructions (Mabtech, Stockholm, Sweden). Briefly, 1 μg/mL of peptides was added to each plate, followed by 5 × 10^5^ PBMCs per well. The plate was incubated at 37 °C for 36 h and the cells were removed by emptying the plate. The reaction was performed according to the instructions provided in the kit. The colored spots were counted and analyzed using an automatic reader (Cellular Technology Ltd., Shaker Heights, OH, USA).

### 2.11. Statistical Analysis

The data were analyzed using Student’s *t*-test and are expressed as the means ± SDs. The differences between the two groups were evaluated by two-way ANOVA (GraphPad Prism; GraphPad Software, San Diego, CA, USA). The differences were regarded as significant if *p* ≤ 0.05.

## 3. Results

### 3.1. Clinical Manifestations of the M3 Strain at Different Doses

**Figure 2:**
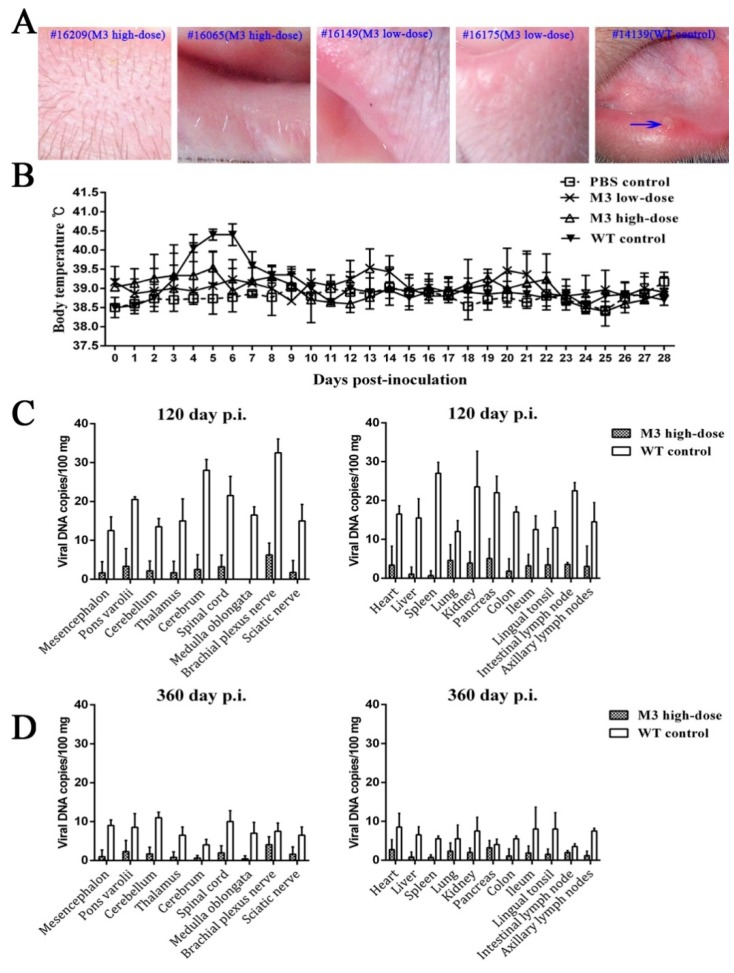
The clinical observations of M3-immunized macaques and the M3 viral DNA copies in various tissues. (**A**) None of the inoculated macaques from the high-dose (#16209 and #16065) and low-dose (#16149 and #16175) groups exhibited symptoms post-immunization compared to the WT control (#14139 was selected as a representative); the vesicle in the lip of #14139 is indicated by the blue arrow. (**B**) The body temperatures of the infected monkeys were measured by the anal route twice each day, post-inoculation. (**C**) M3 distribution in the CNS and other major tissues on day 120, post-immunization, of the high-dose and WT control groups. (**D**) M3 distribution in the CNS and other major tissues on day 360 post-immunization of the high-dose and WT control groups.

Although few studies have reported HSV infection in non-human primates [21,31,32], our previous animal study provided evidence of the infectivity of HSV-1 in rhesus macaques; specifically, infection with the HSV-1 strain F produced oral vesicular manifestation, dynamic viremia, and virus shedding during the acute infectious period [25]. The present study focused on the clinical manifestations in animals inoculated with high and low doses of M3 (5 × 10^5^ TCID_50_ and 5 × 10^4^ TCID_50_ per animal, respectively) for 360 days after inoculation, particularly during the 30-day period. None of the animals inoculated intramuscularly with M3 exhibited clinical symptoms and virus shedding compared to the wild-type strain (Supplementary Table S1; Figure 2A,B). The consistency of the asymptomatic state in the three low-dose and four high-dose macaques supports the low virulence of the M3 strain, which shows the same results as the asymptomatic state; the 100% survival rate was associated with very low viral loads in various organs in mice [33].

### 3.2. M3 Proliferation and Pathogenicity of Inoculated Animals

HSV-1 is a pathogen capable of maintaining a presence in vivo in the infected host [17]. The attenuated HSV-1 strain is of concern due to its potential replication and the formation of certain pathologic lesions in host individuals during asymptomatic states [34]. In our study, the macaques sacrificed 120 and 360 days after M3 inoculation did not exhibit any clinical manifestations compared to the wild-type strain, which supports the very low viral load detected in various tissues from these animals (Figure 2C,D). This characteristic was also observed in our previous mouse study [33]. The results from the immunized macaques sacrificed on day 120 or 360 indicated that less than 10 copies/100 mg of the viral DNA were found by quantitative q-PCR in the main organs (Figure 2C,D). The control macaques infected with the wild-type strain exhibited approximately 10–30 copies/100 mg on day 120 (Figure 2C). To the best of our knowledge, values lower than 10 copies obtained by q-PCR are generally not significant for evaluation [35]. To confirm this finding, the titration of viral infectivity in all the samples was assessed through plaque assays using cultured Vero cells, and similar negative results were obtained. These results support the attenuated phenotype of M3. These results support the attenuated phenotype of M3 that was observed in the mouse model [33].

We further examined the pathogenicity of the M3 strain to identify its attenuated phenotype. Various organs from the immunized macaques sacrificed on days 120 and 360 following M3 inoculation were analyzed histopathologically. No obvious pathological lesions were observed in any organ tissue (Supplementary Table S2), even in the central nervous system (CNS), which usually shows sensitivity to the HSV-1 infection. These observations and the low viral load detected in the various organs support the low pathogenicity of the M3 strain in the inoculated macaques.

### 3.3. Observations of Latent Infection of the M3 Strain in Macaques

**Figure 3:**
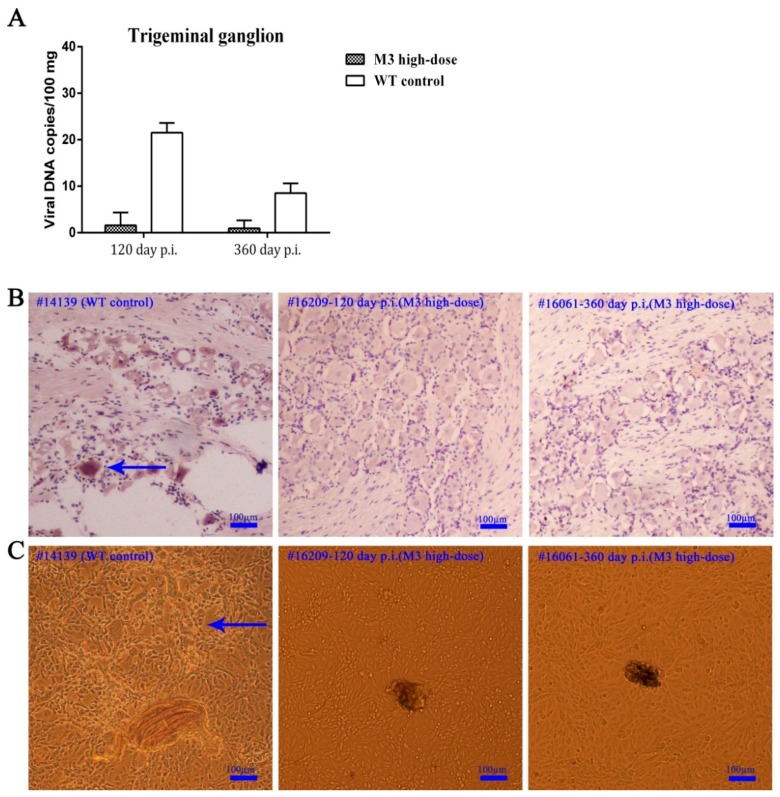
The detection of the M3 latent infection in the TG of inoculated macaques. (**A**) M3 distribution in the TG of macaques from the high-dose group (#16209 and #16061) and the WT control macaques (#14139) on days 120 and 360, post-immunization. (**B**) The in situ hybridization of the TG of WT control macaques (#14139) and macaques from the high-dose group on days 120 (#16209) and 360 (#16061) using a specific *LAT* mRNA probe. Positive in situ hybridization results are indicated by the arrows; scale bars = 100 µm. (**C**) The co-culture of Vero cells with the TG of WT control macaques (#14139) and macaques from the high-dose group on days 120 (#16209) and 360 (#16061). A CPE is indicated by the blue arrows; scale bars = 100 µm.

The viral latency of HSV as an important pathological feature of its infection in humans and animals has been a point of research and is also the key evaluation marker in the study of attenuated and genome-defective vaccines up to this point [33]. Our previous data for the HSV-1 wild-type strain infection in macaques demonstrated the induction of viral latency and the recurrence of vesicular and positive shedding during the first 4 months, post-infection. Positive signals were observed in the TG by in situ hybridization and viral reactivation in cultured cells [25]. The data obtained in the present study suggested that the TG samples from all the immunized individuals sacrificed on days 120 and 360 exhibited very low viral genomic DNA loads compared to the WT control macaques (Figure 3A), which indicates a low level of viral proliferation in this tissue. Notably, none of the TG samples from the immunized individuals sacrificed on day 120 or 360 were positive for lesions (Supplementary Table S2). Furthermore, the in situ hybridization using a specific probe against the *LAT* mRNA sequence suggested a negligible signal in the samples from the individuals sacrificed on days 120 and 360 (Figure 3B). These results suggest a very low probability of M3 latency in macaques, similar to the observations in mice [33]. The homogenized TG from each immunized animal was co-cultured with Vero cells and no viral reactivation was observed. In contrast, the Vero cells that were co-cultured with the TG from WT control macaques exhibited typical CPE (Figure 3C). These results suggested the absence of *LAT* gene transcription in the TGs from animals immunized with M3. However, these data were collected from a limited number of animals (four macaques) and further investigations are needed to confirm our results.

### 3.4. The M3 Strain-Induced Antibody and Cell-Mediated Responses in Inoculated Macaques

**Figure 4:**
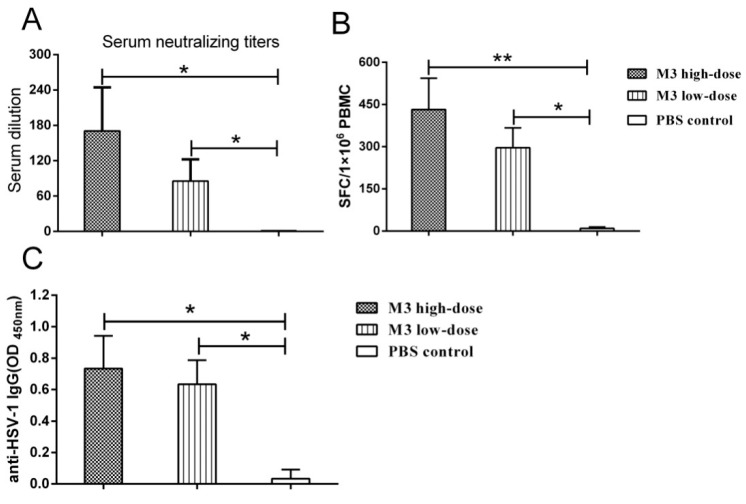
The immunogenicity of the M3-immunized macaques. (**A**) The neutralizing antibody testing of the serum from the high-dose, low-dose, and PBS control groups. (**B**) ELISpot detection of IFN-γ for the analysis of specific T cell responses in the high-dose, low-dose, and PBS control groups. (**C**) ELISA detection of IgG antibodies in serum from the high-dose, low-dose, and PBS control groups. (* indicates *p* < 0.05; ** indicates *p* < 0.01).

An increased immune response was found in the high-and low-dose groups, and the typical neutralizing antibody exhibited a dose effect in serum titrations from all individuals (Figure 4A). Specific T cell responses against HSV-1 were identified in an IFN-γ ELISpot assay using PBMCs collected from all the animals (Figure 4B). These two assays suggested that the M3 induced a specific immunity in the macaques during infection. Robust HSV-1 specific antibody IgG responses were detected in the high- and low-dose macaques but not with the PBS control serum (Figure 4C).

### 3.5. M3-Induced Immunity Defended against the 17^+^ Strain Challenge

**Figure 5:**
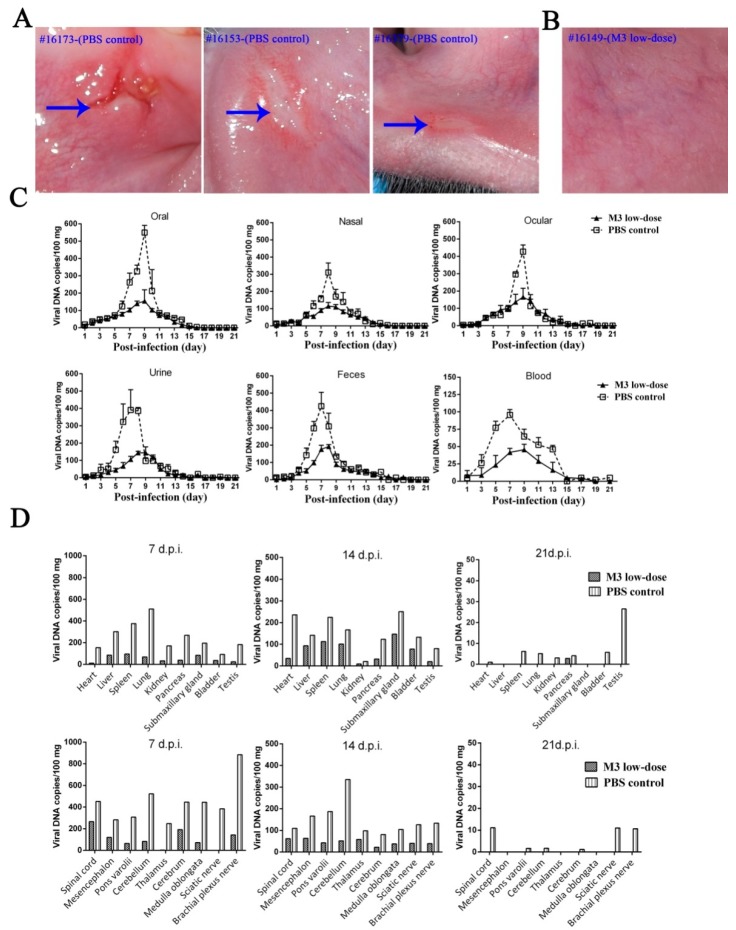
The immune-protective effect observed in M3 low-dose-immunized macaques challenged with the 17^+^ strain. (**A**) Vesicles in the oral mucosa of control macaques (#16173, #16153, and #16379, exhibited on day 5, 8, and 7, post-infection, respectively) are indicated by arrows. (**B**) All the M3 low-dose-immunized macaques were asymptomatic (#16149 was selected as a representative). (**C**) Detection of virus shedding in feces and urine samples and in oral, nasal, and ocular swabs collected for 21 days after the infection. Blood samples were collected every two days for 21 days after the infection. (**D**) Detection of the viral loads in various organs from macaques belonging to the PBS control and M3 low-dose groups collected on days 7, 14, and 21, post-infection.

The above-described observations suggested that inoculation with M3 induced an increased immunity in macaques, but the clinical protective effect of this immunity against the viral challenge was investigated using an immunological study. Animals inoculated with the low-dose of M3 were challenged with the 17^+^ strain 30 days after inoculation, and the protective effects were compared with those observed in the PBS control (three naïve macaques challenged directly with the 17^+^ strain). The clinical manifestations, including oral vesicular lesion, viremia, and viral shedding, were monitored for 21 days. One animal from the low-dose group and one from the PBS control group were sacrificed on days 7, 14, and 21 for further viral detection (Figure 1). The results indicated that the three macaques in the PBS control group exhibited oral vesicular lesions on days 5 to 8 after the viral challenge, whereas the animals in the M3 group were asymptomatic (Figure 5A,B). The detection of viremia and virus shedding at various sites in both groups suggested a significant difference (Figure 5C) and further analysis demonstrated that the viral loads detected in various organs presented significant differences between the challenged animals belonging to the M3 low-dose and PBS control groups at the three different time points investigated (Figure 5D).

Histopathological detection indicated more aggravated inflammatory lesions in some organs, particularly in the CNS tissue (Supplementary Figure S1), from the animals in the PBS control group compared with those from the M3 low-dose group (Supplementary Table S3). However, a trace viral load and slight inflammatory lesions were observed in some organs from the animals in the M3 low-dose group, even if they showed significant differences to the PBS control group. These results increase our understanding of the mechanisms underlying the interaction of the HSV-1 attenuated viral strain with the rhesus macaque.

### 3.6. M3-Induced Immunity Restricted the Entry of the Wild-Type Strain into the TG

**Figure 6:**
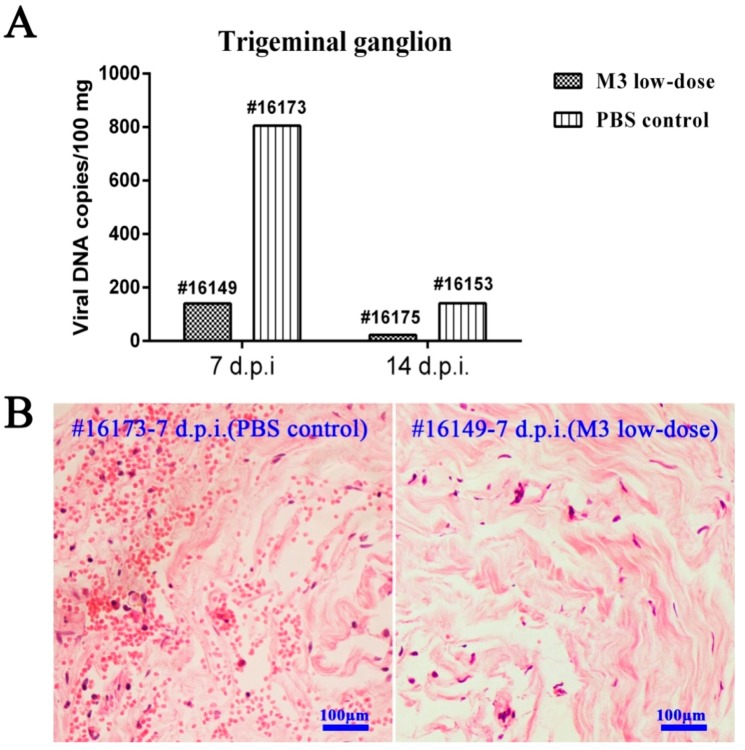
The immunity induced by M3 limits the viral entry into the TG. (**A**) M3 distribution in the TG on days 7 and 14, post-infection; (**B**) Pathological features of the TG of a PBS control macaque (#14139) and an M3-immunized macaque (#16149) on day 7, post-infection; scale bars = 100 µm.

Previous data on HSV infection identified viral latency as an important factor in its pathogenesis [25]. The present study examined the role of immunity in the transmission of the 17^+^ strain into the TG in challenged M3-inoculated macaques. The TG from all the animals was isolated carefully at different time points and q-PCR was used to accurately quantify the viral DNA load. Histopathological observations were used to identify lesions. The results suggested that the PBS control macaques infected with the 17^+^ strain exhibited the highest viral load in the TG 7 days after the challenge. The viral load reached a value higher than 800 copies/100 mg of tissue and decreased to approximately 100 copies/100 mg on day 14 (Figure 6A). The animals in the M3-immunized group had only 150 copies/100 mg of tissue on day 7 and 20 copies/100 mg on day 14 (Figure 6A). Histopathological observations of the TG from the PBS control and M3 macaques revealed more obvious inflammatory lesions in the control group on day 7 (Figure 6B). However, no positive in situ hybridization signal was found in the TGs from either group. To some extent, these results suggested that the M3-induced immunity restricted the entry of the virus into the TG but did not completely block this process. 

## 4. Discussion

Previous studies on HSV, particularly the interaction of HSV with the host, indicated that various encoded viral proteins affect the viral pathogenesis and the antiviral immune response of the host via distinct molecular functions [36,37,38]. These studies indicate that the pathological lesions observed clinically and the immune responses of the host during the disease process are not determined solely by viral replication in tissues [22,39]. Recent studies of HSV attenuated strains demonstrated that certain viral strains with known defective genes encoding *pUs9*, *gE*, *gI*, *gK*, *UL37*, and other gene products enable investigations into the role of the interactions of viral molecules with the host, which impacts the pathological reactions and immune responses of the host in vivo [12,40,41]. These data greatly improved our understanding of viral pathogenesis and the development of treatment drugs or preventive vaccines [40,42]. Notably, the application of the attenuated strain Oka of the varicella-zoster virus, which belongs to the *Alphaherpesvirinae* subfamily of human herpes viruses, along with both HSV-1 and HSV-2, confirmed that the interaction of this attenuated virus and the host produced an effective immunity to defend against the disease [19,43].

Our results using the HSV-1 attenuated strain M3 in macaques contribute to the understanding of HSV-1 pathogenesis and possible vaccine development. Our previous study found that an HSV-1 strain with a mutated gene for the tegument protein *UL7* exhibited low replication in cells and weak virulence in mice [22]. A weaker virulence phenotype was also observed with the M3 strain, which has mutations in the *UL7*, *UL41*, and *LAT* genes [23]. We expected to observe a pathological reactivity and immunity in macaques inoculated with M3 and an attenuated phenotype of strain M3 in macaques. M3 did not induce oral mucosa lesions or histopathological changes in various tissues, and a very low viral load was detected in the tested tissues, even in the CNS and TG, during a long-term period of sensitivity to the HSV-1 infection. These results confirm that modifications to the viral genes *UL7*, *UL41*, and *LAT* reduce the virulence of HSV-1. The data from our work do not support a conclusion that M3 induces complete protection against the viral challenge; instead, they indicate a route for the design of safer vaccine candidate strains. Recently, a report focusing on *UL37*, another tegument protein, identified the protein as being critical for the capacity of the virus to invade the nervous system and constructed an R2 mutant with a deleted *UL37* gene, suggesting a possible way to block the HSV invasion of nervous tissue [41]. The combined findings regarding both R2 and M3 and their capacity to induce immunity in mouse studies [33,41], an increase in a specific neutralizing antibody (Figure 4A), and a cell-mediated response (Figure 4B), especially the protective effects in all the inoculated macaques, do suggest that HSV mutants with attenuated phenotypes by some modified genes might be helpful in vaccine studies. Certainly, a critical limitation of M3 is that it revealed some degree of viral replication in certain organs, including the nervous tissue, of challenged M3-inoculated macaques, and the associated detectable viremia and virus shedding in oral, nasal, ocular, and fecal samples (Figure 5C,D) suggested that further mutations in other genes, such as *UL37*, are needed. Further work based upon the significant difference between the experimental and control groups indicated would focus on the immunity induced by M3 and its inability to completely inhibit the viral proliferation in vivo (Figure 6A) and would seek a new attenuated strain with better immunogenicity.

Previous data explain that this defective immunity is observed in most of the population infected with HSV and might be due to the comprehensive interaction of various viral-encoded molecules with the immune system, particularly, regulators of the innate and adaptive immune responses [44,45,46,47]. For example, the viral-encoded protein Us3 inhibits NF-κB activation and reduces the expression of some cytokines, such as IL-8 [44], and the viral-encoded protein Us5 antagonizes Fas ligand-mediated and granzyme B-mediated CTL-induced apoptosis to inhibit perforin-induced cell death [45]. The important viral immediate-early protein γ34.5 suppresses the PKR/eIF-2a signaling pathway and the IFN-induced anti-viral mechanism and inhibits DC maturation and antigen presentation [46,47]. These data suggest that the establishment of immunity against HSV infection requires systematic immunogenic stimulation of the host with a complete viral antigen and the elimination of viral-encoded molecules obstructing the immune response [48]. An attenuated vaccine might be an appropriate modality for HSV because an attenuated phenotype would not produce pathological lesions in specific tissues, such as the nervous tissues, and the various viral genes that disturb immunity should be modified to produce a systematic immune response. The preparation of this type of viral strain requires further basic studies and technical efforts. However, our study using rhesus macaques demonstrated an attenuated phenotype of M3, and the analyses of the interaction between the virus and its host found that this strain induced a limited immunity. These results will form the basis for further studies and progress toward an attenuated HSV-1 vaccine.

## Supplementary Materials

The following are available online at http://www.mdpi.com/1999-4915/10/5/234/s1. Figure S1: Pathological features M3 low-dose- and PBS-inoculated macaques challenged with wild-type 17^+^ strains; Table S1: Clinical observations of macaques inoculated with the M3 (high and low dose) and PBS groups; Table S2: Histopathological detection of various tissues in the high-dose group; and Table S3: Histopathological detection in tissues in the M3 low-dose and PBS control groups.
