# Supplementary material for: Attenuated Phenotype and Immunogenic Characteristics of a Mutated Herpes Simplex Virus 1 Strain in the Rhesus Macaque
Source: Viruses. 2018 May 2;10(5):234. doi: 10.3390/v10050234 (PMC5977227; doi:10.3390/v10050234)
Supplement: Supplementary file 1 [file viruses-10-00234-s001.pdf]

Table S1 Clinical observations of macaques inoculated with the M3 (high and low dose) and PBS groups

Group	Macaque No.	Diet	Oral vesicle	General symptoms	Viremia	Virus shedding*
**M3 high-dose**	#16209	normal	-	-	-	-
	#16065	normal	-	-	-	-
	#16061	normal	-	-	-	-
	#16137	normal	-	-	-	-
**M3 low-dose**	#16149	normal	-	-	-	-
	#16175	normal	-	-	-	-
	#16319	normal	-	-	-	-
**PBS control**	#16173	normal	-	-	-	-
	#16153	normal	-	-	-	-
	#16379	normal	-	-	-	-

"-": indicates that none of the above symptoms occurred

"*": virus shedding samples from the mouth, nose, eye, feces and urine.

Table S2 Histopathological detection of various tissues in the high-dose group

Tissue	#16209	#16065	#16061	#16137
**TG**	-	-	-	-
**Spinal cord**	-	-	-	-
**Mesencephalon**	-	-	-	-
**Pons varolii**	-	-	-	-
**Cerebellum**	-	-	-	-
**Thalamus**	-	-	-	-
**Cerebrum**	-	-	-	-
**Heart**	-	-	-	-
**Liver**	-	-	-	-
**Spleen**	-	-	-	-
**Lung**	-	-	-	-

Table S3 Histopathological detection in tissues in the M3 low-dose and PBS control groups

	M3 low-dose			PBS Control		
**Tissue**	#16173	#16153	#16379	#16149	#16175	#16319
**Spinal cord**	-	-	-	+++	++	++
**Optic nerve**	-	-	-	++	+	+
**Mesencephalon**	-	-	+	++	+	+
**Pons varolii**	-	+	-	+	+	+
**Thalamus**	-	-	-	+	++	++
**Heart**	-	-	-	++	-	+
**Liver**	-	-	-	++	+	-
**Spleen**	-	-	-	+	++	-
**Lung**	+	-	-	++	++	+

"-": negative.

"+": Slight inflammatory cells infiltration without neural damage.

"++": Slight inflammatory cell infiltration with neural damage.

"+++": Aggravated inflammatory with neural damage.

Supplementary figure legends

Figure S1 Pathological features M3 low-dose- and PBS-inoculated macaques challenged with wild-type 17^{+} strains.

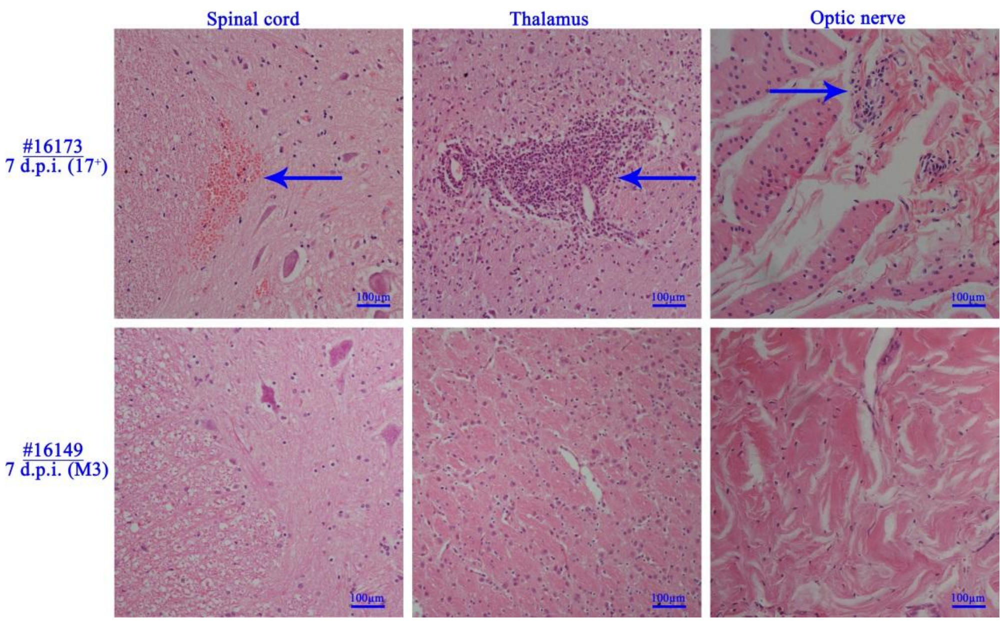

Histopathological detection of spinal cord, thalamus and optic nerve of M3 low-dose macaque(#16149) and PBS control macaques (#16173) , positive pathological change are indicated by arrows, scale bars=100 μm.
